# Laser Technology in Periodontal Treatment: Benefits, Risks, and Future Directions—A Mini Review

**DOI:** 10.3390/jcm14061962

**Published:** 2025-03-14

**Authors:** Dhafer Al Asmari, Ali Alenezi

**Affiliations:** 1 Department of Periodontology and Implant Dentistry, College of Dentistry, Qassim University, Buraydah 51452, Saudi Arabia; 3825@qu.edu.sa; 2 Department of Prosthetic Dental Sciences, College of Dentistry, Qassim University, Buraydah 51452, Saudi Arabia

**Keywords:** safety, lasers, periodontal diseases, periodontal pocket, periodontitis

## Abstract

**Background/Objectives**: The efficacy and safety of laser therapy in periodontal treatment are comprehensively reviewed in this study, focusing on efficacy, safety, patient experiences, and cost-effectiveness. **Methods**: This review encompasses a literature survey, analyzing studies from 2010 to 2024, and a search was conducted in January 2024 across various electronic databases, including PubMed, SCOPUS, EMBASE, COCHRANE library, and Science Direct. The search utilized Mesh terms/keywords such as “Laser therapy”, “Periodontal disease”, “Efficacy”, and “Safety.” **Results**: Out of the initial 884 articles identified, 98 were selected based on their titles and abstracts. After evaluating the full texts and applying the inclusion and exclusion criteria, 16 articles were chosen for the review, meeting the study’s criteria. **Conclusions**: This review identifies gaps in current research and points to emerging trends and potential future advancements in laser therapy, emphasizing the need for standardized protocols, long-term studies, and technological innovations to enhance treatment efficacy and accessibility.

## 1. Introduction

Periodontal disease, a significant public health concern, affects a substantial portion of the global population [1]. According to the World Health Organization, severe periodontal disease, which may result in tooth loss, is found in 10–15% of the global population [2]. The prevalence varies across regions and is influenced by factors such as age, socioeconomic status, and access to dental care [3]. The disease’s impact extends beyond oral health, with links to systemic conditions like cardiovascular disease and diabetes, underscoring its significance in overall health management [4].

Traditional management of periodontal disease primarily involves mechanical debridement, such as scaling and root planing (SRP), coupled with oral hygiene education and, in some cases, adjunctive antibiotic therapy [5]. While these methods are effective in managing mild to moderate cases, they have limitations. Deep periodontal pockets can be challenging to clean thoroughly with mechanical methods, and systemic conditions like diabetes may impair wound healing and response to treatment. Furthermore, the invasive nature of some conventional treatments can lead to patient discomfort and prolonged recovery times [6].

Recent advancements in laser technology have introduced new paradigms in periodontal therapy. Different studies have highlighted the clinical efficacy of low-level laser therapy as an adjunct to non-surgical periodontal treatment [7,8]. Research by Gündoğar et al. [9] and Sumra et al. [10] further explores the role of lasers in both bacterial reduction and hard tissue management, suggesting improvements in clinical outcomes and patient experiences.

Despite promising findings, there remain gaps in the current literature. Most studies have focused on short-term outcomes, with a lack of long-term data on the sustainability of laser therapy benefits. Additionally, there is variability in the types of lasers used and their parameters, which necessitates standardization for clinical practice [11]. Comparative studies between different laser types and traditional therapies in various periodontal disease stages are also limited, which could provide more comprehensive insights [12].

Laser therapy offers several advantages over conventional treatments [13]. It allows for precise targeting of affected areas, potentially reducing damage to surrounding healthy tissues. Lasers can access deeper periodontal pockets more effectively than mechanical tools [14]. The minimally invasive nature of laser therapy can result in reduced patient discomfort and faster healing times [15]. Furthermore, pre-photocoagulation using a diode laser was recommended to decrease the vascular component within the lesion before surgical removal [16]. Additionally, laser therapy shows the potential to enhance tissue regeneration and reduce bacterial loads, leading to improved clinical outcomes [17].

The present mini review aims to provide a comprehensive analysis of laser therapy in periodontal treatment, focusing on efficacy, safety, patient experiences, and cost-effectiveness. By comparing laser therapy with traditional treatments, the study seeks to offer a balanced perspective, highlighting both the benefits and limitations of this novel approach. The goal is to assist dental professionals in making informed decisions based on current evidence, enhancing patient care in periodontology. Furthermore, the review aims to identify gaps in existing research, proposing directions for future studies to advance the field of periodontal therapy.

## 2. Materials and Methods

The search process for this review was carried out according to Preferred Reporting Items for Systematic reviews and Meta-Analysis Protocols (PRISMA) guidelines [18]. To conduct a literature survey on the “Efficacy and Safety of Laser Therapy in Periodontal Treatment”, a search was conducted in January 2024 across various electronic databases, including PubMed, SCOPUS, EMBASE, COCHRANE library, and Science Direct. The search utilized Mesh terms/keywords such as “Laser therapy”, “Periodontal disease”, “Efficacy”, and “Safety.” In addition to the electronic search, cross-references and textbooks were manually searched for relevant articles. The inclusion criteria for the review included articles published in English from January 2010 to January 2024 that specifically addressed the study objectives, i.e., the efficacy and safety of laser therapy in periodontal treatment. The article selection process involved assessing the inclusion and exclusion criteria, as well as conducting a quality assessment. The main exclusion criteria include papers studying other forms of periodontal treatment besides laser therapy. Furthermore, the reference lists of the included studies obtained from the electronic search studies were evaluated for possible additional studies.

## 3. Results

**Figure 1:**
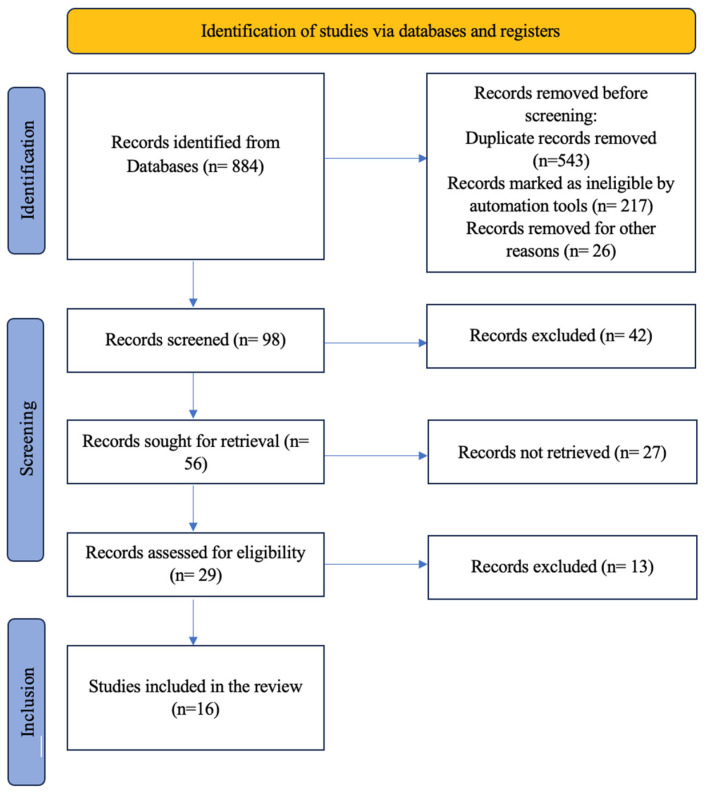
Flowchart showing the step-by-step identification of the studies via databases.

Out of the initial 884 articles identified, 98 were selected based on their titles and abstracts. After evaluating the full texts and applying the inclusion and exclusion criteria, 16 articles were chosen for the review, meeting the study’s criteria (Figure 1). Animal-based studies and narrative reviews on the “Efficacy and Safety of Laser Therapy in Periodontal Treatment” were excluded from the selection process. Table 1 shows summary of selected articles on laser therapy in periodontal treatment.

## 4. Discussion

### 4.1. Historical Perspective of Laser Therapy in Dentistry

#### 4.1.1. Early Development and Adoption

The journey of laser therapy in dentistry began in the mid-20th century, following the invention of the first working laser by Theodore Maiman in 1960 [31]. Early experimentation with lasers in dentistry started shortly thereafter, driven by the potential for precision and minimally invasive treatment. The initial applications were focused on tooth tissue, exploring the potential for cavity preparation and caries removal [32]. However, these early lasers were not immediately embraced in mainstream dental practice due to limitations in technology, high costs, and a lack of understanding of the appropriate applications and safety protocols [31].

#### 4.1.2. Technological Advancements in Laser Therapy

The technological evolution of laser therapy in dentistry has been marked by significant advancements, profoundly enhancing its efficacy, safety, and range of applications [33]. The introduction of specific laser types, such as CO_2_, Nd: YAG, Er: YAG, and diode lasers, tailored for distinct dental procedures, marked a pivotal development in this field. Er: YAG lasers, for instance, gained popularity for hard tissue applications owing to their effectiveness and minimal thermal impact [34]. Further refinements in laser wavelengths and pulse modes enabled more precise control over laser–tissue interactions, significantly reducing collateral tissue damage and improving patient comfort [35]. An important leap was the integration of laser technology with digital imaging and scanning technologies, which has revolutionized treatment accuracy, especially in intricate procedures like periodontal therapy and implantology [36]. Concurrently, technological advancements have led to more compact and portable laser units, along with a reduction in costs, thereby making laser therapy increasingly accessible to a broader range of dental practices [37]. Equally critical has been the enhanced focus on professional training and the development of rigorous safety protocols, which have substantially increased the confidence of dental professionals in employing laser technology [38]. These cumulative advancements have positioned laser therapy as an essential and rapidly growing domain in dentistry, diversifying its applications from periodontal treatment to aesthetic procedures, and significantly contributing to the enhancement of patient care.

### 4.2. Types of Lasers Used in Periodontal Therapy

In periodontal therapy, several types of lasers are employed, each with distinct characteristics and specific applications. The primary lasers used include diode lasers, Nd: YAG (Neodymium-doped Yttrium Aluminum Garnet) lasers, and Er: YAG (Erbium-doped Yttrium Aluminum Garnet) lasers [39]. Understanding the mechanisms and applications of each type is crucial for their effective utilization in periodontal treatment.

#### 4.2.1. Diode Lasers

Diode lasers, operating in the wavelength range of 810 to 1064 nm, function through a mechanism where their light is absorbed by pigments in periodontal tissues and bacteria, resulting in a photothermal effect [40]. This unique property makes them particularly effective for a variety of soft tissue procedures in periodontal therapy. They are extensively used for gingival contouring, providing precise shaping of the gums, and for sulcular debridement, which involves the removal of diseased or inflamed tissue from the periodontal pocket [41]. Additionally, diode lasers play a significant role in the reduction in bacterial populations within periodontal pockets, an essential aspect of treating periodontal diseases [19]. A notable advantage of diode lasers is their hemostatic properties, which help in controlling bleeding during procedures [20]. These have proven effective in treating facial depigmentation and removing soft tissue lesions like pyogenic granulomas [42]. Furthermore, they are known to promote wound healing, making them a valuable tool in periodontal therapy for both their therapeutic and post-procedural benefits.

#### 4.2.2. Nd: YAG Lasers

Nd: YAG lasers, characterized by their operation at a wavelength of 1064 nm, are particularly effective in periodontal therapy due to their high absorption by pigmented tissues and bacteria [21]. This absorption enables efficient tissue ablation and significant bacterial reduction. In the treatment of periodontitis, Nd: YAG lasers are frequently utilized for the debridement of infected tissue within periodontal pockets [22]. Their bactericidal properties are instrumental in controlling and reducing the bacterial load in these areas. One of the key advantages of Nd: YAG lasers is their efficacy in managing deeper periodontal pockets [22]. Their ability to effectively penetrate soft tissue makes them a valuable tool in the treatment of more advanced stages of periodontal disease, where thorough cleaning of deep pockets is crucial for successful therapy. In addition, the Nd: YAG laser was suggested in some clinical situations like lingual frenectomy to be preferred over conventional methods since it can show less bleeding and postoperative pain [43]. This capability, combined with their bactericidal effects, positions Nd: YAG lasers as an essential component in the modern management of periodontal disease [23].

#### 4.2.3. Erbium Family Lasers

Erbium family lasers, including Er: YAG (Erbium-doped Yttrium Aluminum Garnet) and Er: YSGG (Erbium, Chromium-doped Yttrium Scandium Gallium Garnet), are distinguished by their high absorption by water and hydroxyapatite present in both soft and hard dental tissues. This characteristic enables them to achieve efficient ablation with minimal thermal damage, making them highly suitable for a range of periodontal applications [24].

##### Er: YAG Lasers

Er: YAG lasers emit light at a wavelength of 2940 nm. They excel in the removal of calculus and the decontamination of root surfaces, crucial steps in the treatment of periodontal diseases. During periodontal pocket therapy, this laser can potentially provide a more thorough and extensive decontamination and detoxification inside the pockets [17,28,44]. Their precision and minimal invasiveness also extend their use to procedures like gingival retraction, osseous contouring, and bone surgery. Furthermore, because the Er: YAG laser is effectively absorbed by all biological tissues containing water molecules, it can be utilized for both soft and hard tissues [45,46]. The ability of Er: YAG lasers to perform delicate procedures with minimal damage and discomfort positions them as a valuable tool in advanced periodontal therapy, offering enhanced outcomes and improved patient experiences [25]. Meanwhile, the biostimulation effect (photobiomodulation: PBM), which occurs during high-power laser irradiation, could enhance the healing process of periodontal tissues [17,28,44].

##### Er: YSGG Lasers

Er: YSGG lasers operate at a wavelength of 2780 nm. Similar to Er: YAG lasers, they are highly effective in periodontal therapy due to their ability to efficiently ablate both hard and soft tissues with minimal thermal impact. Er: YSGG lasers are particularly noted for their versatility in various dental procedures, including soft tissue surgeries and hard tissue applications such as bone recontouring. Their use in periodontal therapy has shown promising results in reducing bacterial load and promoting tissue regeneration [26]. Regarding the thermal impact, the Er: YAG laser might offer minimal thermal impact compared to the Er, Cr: YSGG laser during irradiation [47].

### 4.3. Efficacy of Laser Therapy in Periodontal Treatment

The evaluation of laser therapy’s efficacy in periodontal treatment is multi-faceted, involving an assessment of clinical outcomes, comparisons with traditional treatments, and analysis of various studies and clinical trials.

#### 4.3.1. Clinical Outcomes of Laser Therapy in Treating Periodontal Disease

Studies have shown that laser therapy can lead to significant improvements in clinical periodontal parameters. These include reductions in probing pocket depth (PPD), gains in clinical attachment level (CAL), and reduced bleeding on probing (BOP) [27]. The effectiveness of laser therapy in these parameters is attributed to its ability to remove diseased tissue, reduce bacterial load, and promote healing [17]. Lasers, particularly Nd: YAG and diode lasers, have demonstrated substantial bactericidal effects in periodontal pockets [28]. This reduction in pathogenic bacteria is crucial in managing periodontal disease, which is primarily caused by bacterial infection. At present, lasers are progressively being incorporated into standard mechanical treatments for periodontal disease, with evidence showing improved wound healing after laser therapy. Some studies have indicated that laser therapy, especially with specific wavelengths like Er: YAG, can enhance tissue regeneration [48,49]. This includes promoting new bone formation, periodontal ligament healing, and reattachment of tissues. In terms of recovery and discomfort, patients undergoing laser therapy often report less pain and faster healing compared to traditional methods. This is largely due to the minimally invasive nature of laser treatment. Nevertheless, the number of clinical studies available is limited, and their beneficial outcomes have not been scientifically confirmed [17].

#### 4.3.2. Comparison with Traditional Treatments

While traditional treatments like scaling and root planning (SRP) are effective, laser therapy offers additional benefits [29]. These include better access to deep periodontal pockets and a higher level of precision in targeting diseased tissues. Furthermore, the treatment of periodontally diseased root surfaces using conventional mechanical methods can be associated with limited accessibility and bactericidal effects within the periodontal pocket [40].

Laser therapy is often used as an adjunct to SRP, with studies showing that the combination can be more effective than SRP alone. This synergistic effect enhances overall treatment efficacy, particularly in managing deeper pockets and more severe forms of periodontitis [6]. In addition, laser therapies can offer noninvasive, bio-stimulatory, and anti-infective approaches to support the conventional treatment methods [50]. It is important to note that laser therapy is not a complete substitute for traditional methods in all cases. Its efficacy can vary depending on the type of laser used, the severity of the periodontal disease, and individual patient factors.

#### 4.3.3. Analysis of Studies and Clinical Trials

There is a wide range of studies with differing designs, laser types, and parameters used, which can make direct comparisons challenging. However, a trend towards positive outcomes with laser therapy is generally observed. Most existing research focuses on short-term outcomes. There is a need for more long-term studies to better understand the sustainability of the benefits offered by laser therapy [51]. A recurring issue in the literature is the lack of standardization in laser application protocols. This variability can impact the comparability of results across different studies. Despite some limitations in the current body of research, the evidence increasingly supports the efficacy of laser therapy in periodontal treatment, particularly when used in conjunction with traditional methods.

### 4.4. Safety and Side Effects

Laser therapy in periodontal treatment is generally considered safe when performed by trained professionals. However, as with any medical procedure, it does carry potential risks and side effects. Understanding these, along with necessary precautions and safety measures, is crucial for optimal patient care.

#### 4.4.1. Potential Risks and Side Effects Associated with Laser Therapy

The potential risks and side effects associated with laser therapy in periodontal treatment, though generally less than traditional methods, are important to consider. Incorrect laser settings or improper handling can lead to tissue damage, including burns to healthy oral tissues, especially when high-intensity lasers are used on delicate areas. Patients might experience pain or discomfort during or after the procedure, but typically to a lesser extent than with mechanical methods. Lasers generate heat, which, if not controlled, can cause thermal injuries to teeth or bone, leading to sensitivity or structural damage [52]. There is also a risk of eye damage for both the patient and the dental professional if proper eye protection is not used, due to the harmful effects of laser light exposure. Additionally, while lasers possess bactericidal properties, their improper use might spread infection or hinder healing. Finally, postoperative bleeding is a risk, albeit less common than in traditional surgery, particularly in patients with bleeding disorders or those on anticoagulant therapy [53].

#### 4.4.2. Precautions and Contraindications

In ensuring the safe application of laser therapy in periodontal treatment, several precautions and contraindications must be strictly adhered to. Only trained and qualified dental professionals should operate laser equipment, as their expertise is key to minimizing risks. A comprehensive review of the patient’s medical history is crucial to identify any conditions that may contraindicate the use of lasers, such as certain systemic diseases, ongoing medications, or allergies. Protective eyewear is mandatory for both the patient and practitioner to safeguard against potential retinal damage from laser exposure. Precise adjustment of laser settings by the specific procedure and tissue type is essential to prevent overheating and consequent tissue damage. Adherence to standard infection control protocols is necessary to avert cross-contamination and the spread of infection. Additionally, laser therapy should be used with caution or avoided entirely in patients with specific conditions like pacemakers, during pregnancy, or those on photosensitive medications, to ensure overall safety and effectiveness of the treatment [54,55].

#### 4.4.3. Patient Safety Measures

Ensuring patient safety in laser periodontal therapy involves a series of crucial measures. Informed consent is fundamental, requiring that patients are fully briefed about the procedure, including its potential risks and benefits, to make an educated decision. Pre- and post-operative instructions should be communicated, guiding patients on expectations and oral care before and after the procedure, thus minimizing complications. During laser therapy, continuous monitoring of the patient is essential to promptly identify and address any signs of discomfort or adverse reactions [25,26,56,57]. Regular post-procedure follow-up appointments are important to monitor healing progress and manage any complications effectively. Tailoring the laser treatment to individual patient needs and conditions not only enhances safety but also improves the effectiveness of the therapy. Additionally, dental practices must have established emergency protocols to efficiently handle any unforeseen situations that might occur during the laser therapy, ensuring patient safety and well-being throughout the treatment process [57,58,59].

### 4.5. Patient Perceptions and Acceptance

#### 4.5.1. Patient Satisfaction and Comfort

Patient satisfaction and comfort are pivotal aspects of any dental treatment, and laser therapy in periodontics has shown promising results in these areas. The minimally invasive nature of laser therapy often leads to reduced pain and discomfort compared to traditional methods like scaling and root planing (SRP). Many patients report a more pleasant experience during laser procedures due to less noise and vibration, which are common discomforts associated with mechanical tools. Additionally, laser therapy typically results in less postoperative pain and swelling, contributing to a quicker and more comfortable recovery [17,60]. The reduced need for local anesthesia in some laser procedures also enhances patient comfort, particularly for those apprehensive about needles. These factors collectively contribute to higher levels of patient satisfaction, as they experience less anxiety and discomfort during and after the treatment. The psychological aspect of undergoing a technologically advanced treatment also plays a role in patient perception, often leading to a more positive overall experience.

#### 4.5.2. Acceptance and Preference Compared to Traditional Methods

The acceptance and preference of laser therapy over traditional periodontal treatments are increasingly evident, especially as awareness and understanding of this technology grow among patients. Many patients are attracted to the idea of a more advanced, less invasive treatment option, perceiving it as a sign of modern and high-quality care. The faster healing times and the reduced risk of complications associated with laser therapy are significant factors influencing patient preference [17,61]. Furthermore, the efficacy of laser treatment in managing periodontal disease, coupled with its aesthetic and comfort advantages, often makes it a preferred choice for patients seeking advanced dental care. However, the decision between laser therapy and traditional methods can also be influenced by factors such as treatment cost, availability, and the specific needs of the patient. As more long-term data on the efficacy and safety of laser therapy become available, patient acceptance and preference are likely to continue evolving, potentially making it a more mainstream choice in periodontal care.

### 4.6. Cost-Effectiveness and Accessibility

#### 4.6.1. Analysis of the Cost Compared to Traditional Treatments

The cost-effectiveness of laser therapy in periodontal treatment is a crucial factor influencing its adoption and patient choice. Initially, laser therapy can appear more expensive than traditional treatments like scaling and root planning due to the high cost of laser equipment and the specialized training required for dental professionals. The advanced technology and the perceived benefits of laser treatment, such as reduced discomfort and faster healing, often contribute to a higher price point. However, when considering long-term outcomes, laser therapy may prove to be cost-effective [17,62,63]. Its efficacy in managing periodontal disease can lead to fewer repeat treatments and lower overall healthcare costs for the patient. Moreover, the minimally invasive nature of laser therapy can result in less time off work and reduced need for postoperative care, which are indirect cost benefits. As the technology becomes more widespread and competition increases, the cost of laser treatment is expected to become more competitive. It is also important to consider the potential cost savings from the preventative aspect of laser therapy, which may reduce the likelihood of more severe periodontal issues and associated treatments in the future [17,64,65].

#### 4.6.2. Availability and Accessibility of Laser Therapy in Different Regions

The availability and accessibility of laser therapy in periodontal treatment vary significantly across different regions. In developed countries, where dental practices are more likely to invest in new technologies, laser therapy is becoming increasingly common. However, in developing countries or remote areas, access to such advanced treatments can be limited. The high initial cost of laser equipment and the need for specialized training are significant barriers to its widespread adoption. Additionally, the lack of awareness among both patients and dental professionals in some regions can influence the availability of laser therapy. As dental technology evolves and becomes more cost-effective, it is anticipated that laser therapy will become more accessible in a wider range of settings. Efforts to improve education and training in laser dentistry, as well as initiatives to reduce equipment costs, can play a significant role in enhancing its global accessibility. Furthermore, with increasing evidence of the benefits of laser therapy, demand from patients may also drive its wider adoption in various regions, leading to improved availability over time.

### 4.7. Future Directions and Research

#### 4.7.1. Emerging Trends in Laser Therapy for Periodontal Treatment

The future of laser therapy in periodontal treatment is marked by several emerging trends that promise to enhance its efficacy and application. One significant trend is the integration of laser therapy with other dental technologies, such as digital imaging and 3D printing, to improve precision and outcomes. Personalized laser treatments based on individual patients’ genetic profiles and specific oral microbiota are also gaining interest, aiming to tailor therapies for maximum effectiveness. Additionally, there is a growing trend towards combining laser therapy with traditional treatments to create a more comprehensive and effective periodontal care approach. The use of specific wavelengths and pulse modes for targeted therapy is being refined, with research focusing on optimizing laser parameters for different stages of periodontal disease. Another exciting trend is the development of portable and more affordable laser devices, making this technology accessible to a broader range of dental practices. These advancements are indicative of a shift towards more patient-specific, technologically integrated, and accessible laser periodontal therapies in the future.

#### 4.7.2. Areas Needing Further Research

Despite advancements, there are areas in laser periodontal therapy that require further research. Long-term clinical studies are needed to better understand the sustainability of laser therapy benefits and its long-term effects compared to traditional methods. Research is also needed to standardize laser protocols and parameters for different periodontal conditions, which would aid in wider adoption and consistent results. The exploration of laser therapy’s impact on systemic health conditions linked to periodontal disease, such as cardiovascular diseases and diabetes, is another area ripe for investigation. In terms of technological advancements, the potential for AI-driven laser systems that can automatically adjust settings based on real-time tissue feedback is an exciting frontier. Development in laser technology that minimizes heat generation and maximizes tissue regeneration capabilities is also anticipated. Additionally, advancements in laser safety and training programs are crucial for ensuring safe and effective treatment delivery. Overall, the future of laser therapy in periodontics holds great promise, with potential advancements that could revolutionize treatment approaches and patient care.

### 4.8. Potential for Technological Advancements

The potential for technological advancements in laser therapy for periodontal treatment is substantial and holds exciting prospects for the future of dental care. With ongoing research and development, we are likely to see innovations that further enhance the precision, effectiveness, and safety of laser applications. One promising area is the integration of artificial intelligence (AI) with laser systems, enabling real-time adjustments and personalized treatment plans based on specific patient needs and tissue responses. Another anticipated advancement is the development of more sophisticated laser devices that minimize thermal damage, thereby reducing the risk of collateral tissue injury and improving patient comfort. Furthermore, advancements in laser wavelengths and pulse modalities are expected to allow for more targeted and efficient treatment of periodontal diseases, including the potential for selectively targeting pathogenic bacteria while promoting tissue regeneration. Portable and cost-effective laser units are also on the horizon, which would make this technology more accessible to a broader range of dental practices and patients. These technological strides, coupled with improved training and safety protocols, are poised to make laser therapy a more integral and effective tool in periodontal therapy.

The primary outcomes of the review indicate that laser therapy in periodontal treatment offers significant benefits, including reduced postoperative pain and swelling, faster healing times, and a reduced risk of complications. These findings are consistent across various studies and clinical trials, demonstrating the efficacy and safety of laser-assisted periodontal treatments. Additionally, the analysis suggests that the long-term use of laser therapy may be cost-effective due to its potential to reduce the frequency of follow-up treatments and minimize the progression of periodontal disease, thereby preventing the need for more invasive and expensive procedures in the future. Based on these outcomes, the objectives were determined to (1) systematically review and synthesize the existing literature on the efficacy and safety of laser therapy in periodontal treatment; (2) highlight the clinical advantages of laser therapy, such as improved patient comfort and faster recovery times; (3) evaluate the cost-effectiveness of laser therapy in the long term; and (4) propose future research directions to address current gaps in knowledge and further optimize the use of laser technology in periodontics.

### 4.9. Strengths and Limitations

The present review has several strengths, including a comprehensive examination of the current literature, highlighting the clinical benefits of laser therapy in periodontal treatment, such as reduced postoperative pain, faster healing times, and potential long-term cost-effectiveness. Additionally, it identifies gaps in existing research and proposes future directions to enhance the use of laser technology in periodontics. However, the review also has limitations, such as potential bias in the selection of reviewed articles and the variability in laser types and treatment protocols across studies, which may affect the generalizability of the findings. Future research should focus on standardized protocols and long-term clinical trials to validate the efficacy and safety of laser therapy in diverse patient populations.

## 5. Conclusions

Laser therapy emerges as a promising adjunct in periodontal treatment, offering advantages like improved clinical outcomes, patient comfort, and efficiency over traditional methods. This review highlights its efficacy and safety while acknowledging the need for standardized protocols and addressing cost and accessibility challenges. The future of laser therapy in periodontics is bright, with potential technological advancements poised to further revolutionize treatment approaches. Continued research and innovation are crucial to fully harness the benefits of laser therapy, ultimately enhancing patient care in dental medicine.

## Figures and Tables

**Table 1:** Summary of selected articles on laser therapy in periodontal treatment.

No.	Authors	Year	Title	Study Type	Key Findings
1	Ren B et al. [7]	2017	A CRISPR/Cas9 toolkit for efficient targeted base editing to induce genetic variations in rice	Experimental Study	Highlighted the clinical efficacy of low-level laser therapy as an adjunct to non-surgical periodontal treatment.
2	Theodoro LH et al. [8]	2021	LASER in periodontal treatment: is it an effective treatment or science fiction?	Review	Demonstrated improvements in clinical outcomes and patient experiences with laser therapy.
3	Gündoğar H et al. [9]	2016	The effect of low-level laser therapy on non-surgical periodontal treatment: a randomized controlled, single-blind, split-mouth clinical trial	Clinical Trial	Suggested improvements in clinical outcomes with the use of lasers in bacterial reduction and hard tissue management.
4	Sumra N et al. [10]	2019	Lasers in non-surgical periodontal treatment–a review	Review	Examined the role of lasers in periodontal therapy, showing potential benefits over traditional methods.
5	De Micheli G et al. [19]	2011	Efficacy of high intensity diode laser as an adjunct to non-surgical periodontal treatment: a randomized controlled trial	Clinical Trial	Demonstrated the effectiveness of high-intensity diode lasers in adjunct to non-surgical periodontal treatment.
6	Aldelaimi TN et al. [20]	2015	Clinical application of diode laser (980 nm) in maxillofacial surgical procedures	Clinical Study	Showed significant results in bacterial reduction and tissue management with diode lasers.
7	Pirnat S et al. [21]	2011	Study of the direct bactericidal effect of Nd: YAG and diode laser parameters used in endodontics on pigmented and nonpigmented bacteria	Experimental Study	Highlighted the bactericidal properties of Nd: YAG and diode lasers.
8	Grzech-Leśniak K et al. [22]	2018	Laser reduction of specific microorganisms in the periodontal pocket using Er: YAG and Nd: YAG lasers: a randomized controlled clinical study	Clinical Trial	Demonstrated significant reduction in specific microorganisms with the use of Er: YAG and Nd: YAG lasers.
9	Zengin Celik T et al. [23]	2019	Clinical and microbiological effects of the use of erbium: yttrium–aluminum–garnet laser on chronic periodontitis in addition to nonsurgical periodontal treatment: a randomized clinical trial—6 months follow-up	Clinical Trial	Showed promising results in bacterial reduction and tissue regeneration with Er: YAG lasers.
10	Abdulsamee N [24]	2017	All Tissues Dental Laser Er: YAG laser—Review Article	Review	Discussed the efficiency of Er: YAG lasers in soft and hard tissue applications.
11	Vinodhini K [25]	2009	Effect of Two Different Laser Treatments on Removal of Root Surface Smear Layer After Root Planing: A SEM Study	Experimental Study	Compared the effectiveness of different laser treatments in removing root surface smear layer.
12	Romanos GE [26]	2021	Lasers in Periodontology. Advanced Laser Surgery in Dentistry	Review	Discussed the various applications of lasers in periodontology and their clinical outcomes.
13	Werner N et al. [27]	2023	Probing pocket depth reduction after non-surgical periodontal therapy: Tooth-related factors	Clinical Study	Investigated the effects of laser therapy on probing pocket depth reduction in periodontal treatment.
14	Mizutani K et al. [28]	2016	Lasers in minimally invasive periodontal and peri-implant therapy	Review	Highlighted the minimally invasive nature of laser therapy and its benefits over traditional methods.
15	Zhao Y et al. [29]	2014	Er: YAG laser versus scaling and root planing as alternative or adjuvant for chronic periodontitis treatment: a systematic review	Systematic Review	Compared Er: YAG lasers to traditional scaling and root planing, showing potential benefits of laser therapy.
16	Sgolastra F et al. [30]	2014	Nd: YAG laser as an adjunctive treatment to nonsurgical periodontal therapy: A meta-analysis	Meta-Analysis	Conducted a meta-analysis showing the effectiveness of Nd: YAG lasers as an adjunctive treatment in non-surgical periodontal therapy.

## Data Availability

Data collected and interpreted in this study are maintained by the authors and can be made available upon request.
